# Factor Structure of the Arthritis Body Experience Scale (ABES) in a U.S. Population of People with Osteoarthritis (OA), Rheumatoid Arthritis (RA), Fibromyalgia (FM) and Other Rheumatic Conditions

**DOI:** 10.2174/1874312900802010064

**Published:** 2008-12-03

**Authors:** J.E.A Boyington, R DeVellis, J Shreffler, B Schoster, L.F Callahan

**Affiliations:** 1 National Institutes of Health, National Institute of Nursing Research, Bethesda, MD, USA; 2 Thurston Arthritis Research Center, University of North Carolina at Chapel Hill, Chapel Hill, NC, USA; 3 Department of Health Behavior and Health Education, School of Public Health, University of North Carolina at Chapel Hill, Chapel Hill, NC, USA; 4 Division of Rheumatology, Department of Medicine, UNC, Chapel Hill, NC, USA; 5 Department of Orthopedics, UNC, Chapel Hill, NC, USA; 6 Department of Social Medicine, UNC, Chapel Hill, NC, USA

## Abstract

**Objective:**

To examine the psychometric properties of the Arthritis Body Experience Scale (ABES) in a US sample of people with osteoarthritis, rheumatoid arthritis, fibromyalgia and other rheumatic conditions.

**Methods:**

The ABES, with the scoring direction modified, was phone-administered to 937 individuals who self-identified as having one or more arthritis conditions based on a validated, US, national survey assessment tool. Descriptive statistics of demographic variables and factor analysis of scale items were conducted. Scale dimensionality was assessed using principal component analysis (PCA) with oblique rotation. Criteria for assessing factors were eigenvalues > 1, visual assessment of scree plot, and structure and pattern matrices.

**Results:**

The predominantly female (74.2%) and Caucasian (79.9%) sample had a mean age of 61.0 ± 13.1 years, and a mean BMI of 30.2 ± 7.1. Major arthritis conditions reported were rheumatoid arthritis, osteoarthritis and fibromyalgia. A three-factor structure with cronbach alpha values of .84, .85 and .53 was elicited, and accounted for 72% of the variance.

**Discussion:**

Compared to the two-factor structure evidenced by the original ABES scale in a sample of UK adults, the data from this sample evidenced a three-factor structure with higher variance. The third factor’s cronbach alpha of .53 was low and could be improved by the addition of salient questions derived from further qualitative interviews with patients with arthritis and other rheumatic conditions and from current literature findings.

**Conclusion:**

The observed psychometrics indicate the scale usefully assesses body image in populations with arthritis and related conditions. However, further testing and refinement is needed to determine its utility in clinical and other settings.

## INTRODUCTION

Arthritis and related rheumatic diseases encompass more than 100 conditions, and in addition to pain and inflammation, are often associated with deformity and body disfigurement [1, 2]. Body disfigurement has been linked to depression, poor self esteem, negative body image perceptions, and unhealthy health behaviors [3-5]. Research about body image in people with arthritis and other rheumatic conditions is limited, however, findings indicate that concerns about physical appearance are common [6].

Conceptually, body image is defined as a multi-dimensional construct including affective (feelings and behaviors toward the body, its processes and functions) and perceptual (appearance, size and shape) components [7, 8] Current body image instruments measure either one or both of these dimensions [9, 10] which are synonymously described in the literature as “body concept” “body scheme” “body satisfaction” “body identity” and “body experience” among others [11].

Historically, body image measures were designed to assess the construct in people with obesity or eating disorders [7, 10, 12], and the majority of instruments were therefore specific to these conditions. Consequently, with respect to arthritis and related rheumatic conditions, there are currently “no well validated measures of appearance that have been extensively used with patients with rheumatic diseases” [6]. This is of concern since many types of arthritis and rheumatic conditions affect physical appearance, and physical appearance concerns are associated with negative body image perceptions, engagement in self-management, and health outcomes [7, 13]. Additionally, studies about body image in these populations are limited and also currently difficult to conduct because of the lack of validated instruments.

The need to change this situation has led to the development of an arthritis-specific body image instrument recently featured in a publication of the *Scandinavian Journal of Rheumatology*. Showcased in a study of 40 people with ankylosing spondylitis was an instrument titled the Arthritis Body Experience Scale (ABES) [14]. The ABES was developed in England in a sample of 119 people with various types of arthritis [15]. Using patient interviews, current literature findings and expert opinions, a 15-item instrument was reportedly generated, and through factor analysis reduced to a 9-item, 2 subscale instrument which correlated with a validated, generic body image instrument, the Body Satisfaction Scale (BSS) [16]. Since its development in the original study, the 9-item ABES has however been featured in only the one study noted above [14] and was limited to one type of arthritis, specifically, ankylosing spondylitis. In that study, correlations with measures related to body image were reported, but no findings of the psychometrics were presented. As such the dimensionality and performance of the ABES in other populations of people with arthritis and other rheumatic conditions is still unknown. In light of the scarcity of measures of appearance that are specific for arthritis and arthritis-related disease populations, it is important that further assessments of the performance of the ABES as a tool for body image assessments be conducted.

This paper presents findings of a study the “Variations in Body Image” (VBIA) study which purposed to assess variations in body image perceptions, pain and limitations in activity of daily living among people with arthritis and related rheumatic conditions. Since, body image was a major parameter of interest of this study, there was a need to find valid instruments for assessing this factor and hence the assessment of the ABES, whose findings are being reported in this paper. Details of both this study and the parent study in which it was nested are described in detail in the methods section.

## MATERIALS AND METHODOLOGY

### Sample

In 2001 family practice settings in urban and rural communities across the state of North Carolina were recruited into a unique, state-wide, practice based research network, officially titled the North Carolina Family Medicine Research Network (NC-FM-RN) [17]. Adult patrons of the family practice settings were recruited to form a research cohort, which was enriched with new participants in 2004 and 2005. Currently the network includes twenty-five family practice settings and more than 4000 individuals.

The parent study of this VBIA study was initiated in 2004. At that time 2420 individuals from the NC-FM-RN cohort were recruited and administered a phone survey which assessed individual and community determinants of chronic disease outcomes. In 2006 a second follow-up phone survey was embarked upon for the parent study and the VBIA study was then designed and inserted into the parent study. From the eligible pool of 2420 initial participants in the parent study’s first survey, 1541 individuals were eligible for the second survey and were administered the survey in 2006. From that pool of 1541, a total of 937 individuals self-identified as having one or more arthritis and arthritis-related conditions. This group of 937 served as the sample for the VBIA study and was administered the ABES instrument. The total participation rate for the parent study in 2006 was 63.7%. All research protocol and methods for the parent study, this sub-study, and the NC-FM-RN were reviewed and approved by the University of North Carolina Medical Institutional Review Board.

### Measures

In addition to the ABES, which was the instrument of interest in this VBIA study, socio-demographic measures were also assessed and included age, race, gender, education, body mass index (BMI) and income levels. Age was gathered using date of birth and was converted to years during the analysis. Race was categorized as non-Hispanic white, non-Hispanic black or other. Education was collected in several levels but was subsequently categorized as 0 = less than high school, 1 = high school and 2 = greater than high school. Using the formula BMI = weight in kilograms/(height in meters)^2^, self-reported weight in pounds was converted to kilograms, and height in feet and inches, to meters, to determine individuals’ BMI. Income was measured in the following 6 general categories: 1 = less than $15K; 2 = ≥ $15K and < $30K; 3 = ≥ $30K and < $45K; 4 = ≥ $45K and < $60K; 5 = ≥ $60K and < $75K and 6 = > $75K. Arthritis status was determined using the 2003 arthritis module of the Behavioral Risk Factor Surveillance Survey (BRFSS). The BRFSS is a United States Centers for Disease Control and Prevention (CDC) validated survey tool used by state health departments to monitor health risk and health behaviors. The 2003 arthritis module included the questions “Have you EVER been told by a doctor or other health professional that you had [*specific arthritis*]? Affirmative responses were further validated by asking the participants to specify the arthritis type using response options that included osteoarthritis (OA), rheumatoid arthritis (RA), fibromyalgia (FM), lupus, and gout among others [18].

### ABES Instrument

The original ABES is a composite of two subscales individually titled *Body Totality *and* Body Self Consciousness. *The instrument consists of 9 items anchored on a 10-point Likert scale. The anchor responses are 1= ‘strongly disagree’ and 10 = ‘strongly agree’. The first 5 items are grouped under the *Body Totality* section and the next 4 items under the *Body Self Consciousness *section. As listed the *Body Totality *section includes the items:

1. **I am happy with my body-**
2. **I am happy with my posture–**
3. **I am happy with the way I walk-**
4. **My body is physically attractive-**
5. **I am concerned with the physical fitness of my body-**
  The remaining 4 items under the *Body Self Consciousness* section are
6. **I am self-conscious about my body-**
7. **I am self-conscious about the parts of my body affected by arthritis that are visible to others-**
8. **I wear particular clothing to hide certain parts of my body affected by arthritis-**
9. **I am embarrassed about the parts of my body affected by arthritis-**

## DATA COLLECTION

In both the original and the only other study cited to have used the ABES, the administration method was reported as paper and pencil format. However, in our study, a phone format was used because the parent study, in which this study was nested, was an existing and on-going phone survey project. Therefore, to feasibly administer the scale, the mode of administration had to be adapted. To facilitate administration of the ABES and all other study measures, a professional university-managed phone survey center (The Survey Research Unit - University of North Carolina-Chapel Hill), was contracted. Pilot testing of the scale was conducted by phone and results indicated the need for a few modifications. First, the scale’s introductory statements were written to indicate that the scale was a body experience scale being administered by an interviewer. Next, the word “posture” in item 2 was expanded to include the phrasing, “the way you carry yourself”. No new questions or items were added to the scale and no other changes in the wording of the questions were made. Finally, the direction of scoring was reversed to correspond to the direction of scoring of other scales in the parent study’s survey which also used ‘agree’ and ‘disagree’ as anchors. Thus the value ‘1’ which initially represented the anchor ‘strongly disagree’ was changed to represent ‘strongly agree’ and the value ‘10’ which represented ‘strongly agree’ was changed to represent ‘strongly disagree’. Prior to the conduct of the main phone survey, training was provided to the interviewers in accordance with the changes indicated. Scale scoring was accomplished by summing the value of each response in each subscale to generate separate scores for the *Body Totality and Body Self Consciousness* sections. Because of the reversed scoring adopted, low scores, (high agreement) on the *Body Totality* and high scores (high disagreement) on the *Body Self Consciousness *scale were indicative of positive body experience or positive body image. In accordance with current usage in the literature and for the purpose of discussion the terms ‘body image’ and ‘body experience’ are used interchangeably in this article.

## ANALYSIS

Descriptive overall group and subgroup analyses were conducted to characterize the sample and determine its demographic profile relative to age, gender, income, and race. To determine the scale’s properties, factor analysis was conducted using SPSS 14.6 (2007, Chicago, USA). Criteria for evaluating factor presence and scale structure included latent root method, i.e. eigenvalues > 1; visual assessment method using scree plot; and assessment of factor loadings on the pattern and structure matrices. Principal components analysis (PCA) was used to factor analyze the data. Of the 937 individuals who self-identified as having arthritis and related conditions and were administered the ABES, 899 had complete data and served as the sample on which factor analysis was conducted for the overall group. Oblique rotation was selected as the rotation method because the inter-item correlation matrix indicated numerous, strong inter-item correlations for most items, and also because this was the rotation method used, with PCA, by the original study. Kaplan Meyer Olkin measure of sampling adequacy and Bartlett test of sphericity were also conducted to ascertain data sampling adequacy and the appropriateness of using factor analysis for the data.****Further analyses were also conducted on the three major rheumatic conditions or subgroups (rheumatoid arthritis, fibromyalgia and osteoarthritis) using mutually exclusive samples. The same factor analysis criteria were applied for all three subgroups.

## RESULTS

The study’s overall sample was predominantly female (74.2%) and Caucasian (79.9%), with a mean age of 61.0 ± 13.1 years (Table **1**). The mean BMI was 30.2 ± 7.1 and 16.0% had less than a high school education. Almost one-quarter (24.5%) had an income of less than $30,000.00. Over half (51.3%) reported osteoarthritis (OA), 12.6% reported fibromyalgia and 23.3% reported Rheumatoid Arthritis. Additionally, over 46.8% reported bursitis or tendonitis, 21.3% reported carpal tunnel, 14.4% reported gout and 11.8% reported other types of arthritis (Table **1**). Many individuals reported more than one of these conditions. The proportion of people who reported Rheumatoid arthritis, Fibromyalgia, and Osteoarthritis as mutually exclusive primary conditions were, 23.3%, 9.5% and 34.3%. Compared to the main sample, the 38 individuals with missing data had proportionately less female (69.2%), was slightly older (70.9 + 14.0) and had a mean BMI of 29.0 ± 7.9. Additionally, 18 individuals (45%) from this group did not report their income levels. But for those who reported income, 40% received less than $30,000 annually compared to 24.5% of the overall sample.

### Overall Group’s Factor Analyses Findings

**Fig. (1):**
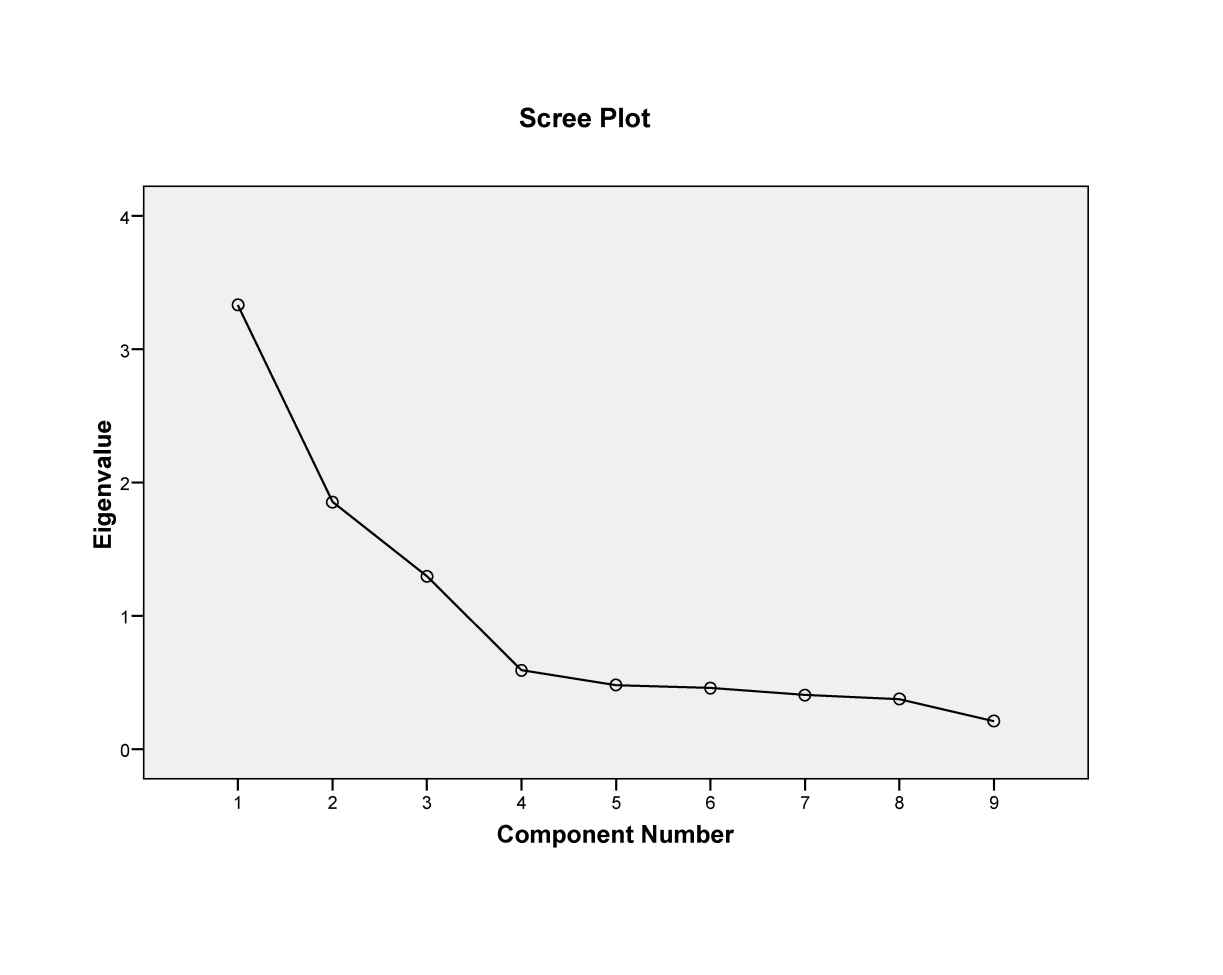
Scree plot of 3-factor structure of the overall sample.

Correlation matrices from the factor analysis of the data of the 899, indicated that all but 2 of the inter-item correlations were significant (p < 0.05). Kaiser Meyer Olkin measure of sampling adequacy for the dataset was = 0.77; and Bartlett’s test of sphericity was significant at p < 0.001 (Table **2**). Initial principal components analysis with direct oblimin rotation indicated a three-factor structure, the first of which contained 4 items, (#1-4). Factors 2 and 3 contained three items, (#7-9), and two items, (#5 and 6), respectively. Evaluation of the structure matrix revealed the item loadings for factor 1 ranged between 0.81-0.84, for factor 2, between 0.80-0.91, and for each of the two items in factor 3, 0.83 and 0.82 respectively (Table **3**). The two items in factor 3 did not load above 0.24 on either factor 1 or 2. The alpha coefficients (α) for the three factors identified were: α = 0.84 (factor 1), α = 0.85 (factor 2), and α = 0.53 (factor 3). The total variance explained by this three-factor structure was 72% (Table **4**). Examination of the scree plot (Fig. **1**) also indicated a three-factor structure. The component correlation matrix indicated negligible correlation between factor 3 and the other 2 factors (Table **5**).

Following the initial analysis an attempt was made to force the two items in factor 3 into a two-factor model to conform to the findings of the original study. This resulted in reduced alphas for the resulting two factors, (subscales), and an overall reduction in the scale variance from 72% to 57.6%. Thus comparing the findings of the two analyses, the three-factor structure explained more variance and was therefore accepted as valid for this data.

### Subgroup Factor Analyses Findings

The sample of people who had RA as the primary diagnosis was 219. Factor analysis of their data revealed a three factor structure with loadings of 0.83 to 0.88 for factor 1(4 items) 0.77 to 0.92 for factor 2 (3 items) and -0.78 to -0.83 for factor 3 (2 items). For the 89 people with fibromyalgia as the primary diagnosis, the same three-factor structure was also evidenced, however the directions for items in factor three were positive instead of negative. Loadings for factor 1 were 0.69 to 0.84 (4 items), factor 2, 0.87 to 0.91(3 items) and factor 3, 0.84 and 0.88 (2 items). Similarly, for the 322 people who had OA as their primary diagnosis factor analysis of their data also revealed a three factor structure with the same number of items as observed as in the RA and FM data. Again the direction of the loadings for the two items in factor 3 was positive as opposed to negative as observed in the data for RA. Specifically for the OA data, factor 1 had loadings between 0.82 to 83 (4 items), factor 2 had loadings between 0.81 to 0.91(3 items) and factor 3 had loadings of 0.83 for each of its two items. The total variance explained by the three factors for the data from the three samples, was 74.7%, 72.2% and 72.5% for RA, OA and FM, respectively. Hence the factor structure, loadings and variance explained in the subgroup analyses were similar to that of the overall sample. Analyses for other rheumatic conditions were prohibited by small sample sizes.

Noting the posited relationship between obesity and arthritis, correlation analyses were conducted for BMI and arthritis status. The mean BMI for of the three primary conditions were similar to that of the overall group. Specially, for OA, BMI = 29.92 ± 7.03; RA, BMI = 30.23 ± 7.70 and FM, BMI = 30.81 ± 8.02. These means were not significantly different. Additionally, correlation of BMI with arthritis status indicated no significant relationship for any of the three primary conditions. Correlation statistics and p values for each were: RA, r = 0.01, p =0.74; OA, r = 0.03, p = 0.39; and FM r = 0.03, p = 0.42. Finally, the relationship between BMI and each of the items in the ABES was also examined and statistically significant correlations were evident for all (p < 0.05) except for item # 5, p = 0.39.

## DISCUSSION

This study purposed to confirm the factor structure of a slightly modified version of the ABES in a U.S sample of people with arthritis and related conditions. Compared to the original UK study’s findings of two factor structure, this study observed a three-factor structure. The two factors of the original ABES were titled the *Body Totality* and *Body Self Consciousness subscales*. The α coefficient presented for the *Body Totality* subscale, which included the first 5 questions, was = 0.72 and the α coefficient for the *Body Self Consciousness*, which included the last 4 questions, was 0.84. The overall variance reported for these two factors (subscales) was 52%. For the total sample of 899, our study elicited a three-factor structure with α coefficients of 0.84 (factor 1), 0.85 (factor 2) and 0.53 (factor 3) and a total variance of 72%. Similar findings were also evident in the subgroup analyses done for RA, OA and FM. The first factor produced by our findings for the overall group, contained items 1-4 in the ABES and essentially corresponds to the *Body Totality* subscale. However, it had a higher α coefficient of 0.84. Similarly, our second factor which contained items 7-9, corresponds to the *Body Self Consciousness *subscale and it had an α coefficient of 0.85. The third factor which emerged had two items, each of which had loadings > 0.82 on the factor, and no loading higher than 0.24 on the other two factors. The loadings exhibited by the two items in the third factor indicated that these two items were tapping on a construct separate from those in factors 1 and 2. The specific questions tapped were:** item #5. *I am concerned with the physical fitness of my body* and item #6.* I am self-conscious about my body*. **Given that item #5 consistently loaded higher than item #6 and was concerned with assessing physical fitness perception the third factor was labeled “*Body Physical Function” to reflect its tapping on physical fitness and self-consciousness about physical fitness*. As stated we attempted to force the two items in factor 3 into a two-factor model similar to that evidenced by the original ABES, but this resulted in a reduction of the reliabilities of the factors (subscales) and a reduction in the overall variance to 57.6%. Combined with the findings from the correlation matrix, we conclude that the two items in factor 3 are salient to this scale but are tapping a construct different from those tapped by the other factors. Furthermore, the low reliability of 0.53 evidenced by factor 3 indicates that the two items are not completely reflecting the construct in factor 3 and thus addition of other relevant items are needed. Salient items for factor 3 could be determined through a review of the current literature, consultation with rheumatology experts and clinicians, and qualitative inquiry of people with arthritis.

Compared to the original study, differences observed in this study could possibly be attributed to differences in the mode of administration. Whereas the original ABES was administered in a paper and pencil format, this study had to use a phone-interview format. We perceive that participants could have indicated different responses if they had had the option to revisit previously answered questions on paper or could see all of their responses as a whole. Secondly, it is also possible that reversing the direction of the scoring may have been confusing for some participants and inadvertently led to the selection of values which were not reflective of their level of agreement on the items tested. Feedback on the scale’s administration suggests that people generally associated the value ‘10’ with “strongly agree”, and when reversed as was done in this study, may have caused some difficulty. However the similarity in item distribution and loadings evidenced by this data as compared to the original study suggest that mis-selection was not a problem.

For both the original and the current study, the percent of women (78% *vs* 79.9%) and the mean age (59.0 and 61.0 years old) were similar. However, there were also several dissimilarities which could account for the observed differences. First, the original study included 119 people with the following distribution of arthritis types: Rheumatoid Arthritis (24%), Osteoarthritis (35%), ankylosing spondylitis (8%) and other arthritis (osteoporosis, back pain, psoriatic arthritis, etc (33%)). Our study had a comparatively larger sample of respondents (899), was more racially diverse ((17% minorities *vs* 2%) (3% of the people did not report race)), and had larger proportions of people with various types of arthritis and arthritis related conditions (Table **1**). Comparatively speaking, the largeness and heterogeneity of our sample provides more robust information about the psychometrics, performance and dimensionality of the ABES in people with arthritis and related conditions in the US. However, further investigations are needed to confirm the factor structure in other populations with arthritis and other rheumatic diseases.

An issue of interest to the construct being measured by this scale is the fact that both studies had a large percentage of women. It is reported that women are generally more conscious about their body image [10]. Therefore, it is possible that for this group, where a high proportion of women over 40 were present, body self-consciousness due to mid-life weight gain may have been present. However, correlation analyses indicated no significant findings between BMI and arthritis status for the overall group or the subgroups examined. Significant correlations for BMI and all but one of the ABES items were however observed. This suggests that BMI is related to all but one of the specific questions in the ABES but not to arthritis status. Given the relationship between obesity and arthritis, this observation is significant especially in light of the fact that this primarily Caucasian, female sample had a mean age of 61, an overall BMI of 30 (obese), and obesity is associated with body image dissatisfaction [7, 8, 10]. Further clarification of the interaction between obesity and these conditions is needed to justify the use of this scale exclusively for the assessment of body image in these populations.

One potential limitation of this study is that disease status was self-reported and therefore could not be confirmed. It is possible that this could have limited the validity of the findings. However, we feel that this is not the case and does not affect the outcomes since we used a nationally validated survey questionnaire (BRFSS-arthritis module) to assess disease status. In addition, affirmative responses of current disease status were further validated in the survey by having participants specify the type of condition they had. Thus participants were less likely to make up their disease status.

Another possible limitation of the findings from this study is the inclusion of people with fibromyalgia. Fibromyalgia is technically not a type of arthritis. However, it is classified as a rheumatic condition because of the similarity of its symptoms to arthritis. With respect to this study, the ascertainment of arthritis status was done by the use of a nationally validated instrument which included fibromyalgia as one of the response options, and so our study also included fibromyalgia as a related condition. It is possible that a significant proportion of people with fibromyalgia may have had body image issues related to indirect, disease-related causes of body changes such as weight gain due to inactivity or to certain medications used [19], and this could have influenced their body experiences. Analysis of the data for fibromyalgia participants did not show a difference in the factor structure when compared to the overall group’s findings. This suggests that their body image, though not resulting from overt physical damage due to disease activity, for example joint damage, is yet still similar to that of other arthritis populations. We however, did not assess whether people with fibromyalgia were currently taking medications associated with weight gain or had experienced weight gain due to inactivity. We however, believe that valuable information was gained from including this condition, because it is characterized by much of the same features of pain, activity limitations and subsequent weight gain which affect body image in people with arthritis. We therefore perceive that the inclusion of people with fibromyalgia provides further information on the utility of the ABES for other rheumatic conditions which do not have overt and direct disease-induced body changes. Our findings also indicated that though the RA sample evidenced the same factor structure as the other subgroups and the overall group, the direction of the loadings in factor 3 was different. This difference in direction suggests that testing in other groups with specific types of arthritis and rheumatic conditions that affect body image is warranted specifically so that similarities in aspects of body image manifested in people with different causes of body changes and types of arthritis can be ascertained.

## CONCLUSION

In this paper, we present the first analysis and psychometrics of an arthritis-specific body image instrument, the ABES, in a U.S population. Our study found differences in the factor structure of the scale as compared to the original study and suggests that further testing to ascertain performance in different arthritis and arthritis-related populations be pursued. The study documents that the ABES does measure aspects of body image important to people with arthritis and other rheumatic conditions and finds that it can be effectively used as a phone-based instrument. The consistency in the factor structure evidenced in the overall and subgroup analyses indicate that body image is an important concern for people with arthritis and related conditions. However, the differences observed in this sample’s factor structure compared to the original ABES sample, indicate that aspects of body image may be different across rheumatic conditions and populations. Nevertheless, body image is an important and clinically relevant variable for people with arthritis and other rheumatic conditions and the lack of validated scales of appearance should not preclude the examination of body image concerns in these populations. Clinicians could assist people with arthritis and related conditions by exploring patients’ perceived impact of their condition on their body function, body shape and body attractiveness, and where warranted, direct them to strategies that facilitate positive body image and improve quality of life.

## Figures and Tables

**Table 1:** Frequency Distribution of Demographic and Other Characteristics of the Study Sample (N=937)*

Characteristics	Percent (%)
Females	74.2
Caucasians	79.9
Income < $30,000	24.5
Less than HS education	16.0
Osteoarthritis	51.3
Rheumatoid arthritis	23.3
Fibromyalgia	12.6
Bursitis/Tendonitis	46.8
Carpal Tunnel	21.3
Gout	14.4
Other arthritis	11.8
	**Mean ± SD**
BMI	30.2±7.1
Age	61±13.1

* Complete case analysis only. Many individuals reported multiple arthritis conditions.

**Table 2:** KMO and Bartlett's Test Statistics of 3-Factor Structure of the Overall Sample

Kaiser-Meyer-Olkin Measure of Sampling Adequacy	.77
** Bartlett's Test of Sphericity**	
Approx. Chi-Square	3041.57
df	36
Sig.	.000

**Table 3:** Structure Matrix of the 3-Factor ABES Scale of the Overall Sample

Items Components			
	**1**	**2**	**3**
1. I am happy with my body	**.839**	-.233	.042
2. I am happy with my posture	**.813**	-.280	.062
3. I am happy with the way I walk	**.820**	-.329	.066
4. My body is physically attractive	**.814**	-.125	.033
5. I am self-conscious about the parts of my body affected by arthritis that are visible to others	.189	-.061	**.825**
6. I am concerned with the physical fitness of my body-	-.096	.238	**.821**
7. I am self-conscious about my body	-.208	**.803**	.257
8. I wear particular clothing to hide certain parts of my body affected by arthritis	-.248	**.907**	-.009
9. I am embarrassed about the parts of my body affected by arthritis	-.305	**.904**	.031

Extraction Method: Principal Component Analysis. Rotation Method: Oblimin with Kaiser Normalization. Bold Numbers Indicate Items’ High Loadings.

**Table 4:** Factor Loadings and Total Variance for 3-Factor Model of the Overall Sample (Total Variance Ex-plained)

Component	Extraction Sums of Squared Loadings			Rotation Total
	Total	% of Variance	Cumulative %	
1	3.33	37.03	37.03	2.94
2	1.85	20.58	57.61	2.60
3	1.30	14.40	72.00	1.43

Extraction Method: Principal Component Analysis.

**Table 5:** Component Correlation Matrix of the 3-Factor Structure of the Overall Sample

Component	1	2	3
**1**	1.000	-.289	.059
**2**	-.289	1.000	.096
**3**	.059	.096	1.000

Extraction Method: Principal Component Analysis.

Rotation Method: Oblimin with Kaiser Normalization.
